# Adaptive Feeding Robot With Multisensor Feedback and Predictive Control Using Autoregressive Integrated Moving Average–Feed-Forward Neural Network: Simulation Study

**DOI:** 10.2196/69877

**Published:** 2026-01-27

**Authors:** Shabnam Sadeghi-Esfahlani, Vahaj Mohaghegh, Alireza Sanaei, Zainib Bilal, Nathon Arthur, Hassan Shirvani

**Affiliations:** 1 Faculty of Science & Engineering, Anglia Ruskin University, Bishop Hall Lane, Chelmsford, CM1 1SQ, United Kingdom, 44 07944281517

**Keywords:** assistive technology, ARIMA, autoregressive integrated moving average, FFNN, feed-forward neural network, feeding robotics, forecasting, motor impairment, time series analysis, personalized assistance

## Abstract

**Background:**

Eating is a primary daily activity crucial for maintaining independence and quality of life. Individuals with neuromuscular impairments often struggle with eating due to limitations in current assistive devices, which are predominantly passive and lack adaptive capabilities.

**Objective:**

This study aims to introduce an adaptive feeding robot that integrates time series decomposition, autoregressive integrated moving average (ARIMA), and feed-forward neural networks (FFNN). The goal is to enhance feeding precision, efficiency, and personalization, thereby promoting autonomy for individuals with motor impairments.

**Methods:**

The proposed feeding robot combines information from sensors and actuators to collect real-time data, that is, facial landmarks, mouth status (open or closed), fork-to-mouth and plate distances, as well as the force and angle required for food handling based on the food type. ARIMA and FFNN algorithms analyze data to predict user behavior and adjust feeding actions dynamically. A strain gauge sensor ensures precise force regulation, an ultrasonic sensor optimizes positioning, and facial recognition algorithms verify safety by monitoring mouth conditions and plate contents.

**Results:**

The combined ARIMA+FFNN model achieved a mean squared error (MSE) of 0.008 and an *R*^2^ of 94%, significantly outperforming the standalone ARIMA (MSE=0.015; *R*^2^=85%) and FFNN (MSE=0.012; *R*^2^=88%). Feeding success rate improved from 75% to 90% over 150 iterations (*P<.*001), and response time decreased by 28% (from 3.6 s to 2.2 s). ANOVA revealed significant differences in success rates across scenarios (*F*_3,146_=12.34; *P*= .002), with scenario 1 outperforming scenario 3 (*P*=.030) and scenario 4 (*P*=.010). Object detection showed high accuracy (face detection precision=97%, recall=96%, 95% CI 94%-99%). Force application matched expected ranges with minimal deviation (24 [1] *N* for apples; 7 [0*.*5] *N* for strawberries).

**Conclusions:**

Combining predictive algorithms and adaptive learning mechanisms enables the feeding robot to demonstrate substantial improvements in precision, responsiveness, and personalization. These advancements underline its potential to revolutionize assistive technology in rehabilitation, delivering safe and highly personalized feeding assistance to individuals with motor impairments, thereby enhancing their independence.

## Introduction

Eating unaided remains impossible for millions of people with upper-limb impairments, turning every meal into a reminder of lost autonomy. Demographic change will magnify this challenge. By 2050, 1 in 6 individuals will be aged over 65 years, up from 1 in 11 in 2024 [1]. Aging correlates with disability, and about 1 billion people already live with an impairment, a figure set to rise as populations gray [2]. The result is a rapidly growing cohort that needs daily feeding assistance, further straining care networks that are already under pressure [1,3]. Assistive devices are critical, especially in low-resource settings where professional carers are limited. In the United Kingdom, for example, many adults aged over 75 years live alone and receive only brief daily visits, leading to malnutrition and declining health [4,5].

Robotic feeding assistants offer a promising solution, demonstrating consistent patience, adaptability, and precision. By tailoring actions to user preferences, they can provide a personalized and empowering dining experience [6,7].

Several commercial and noncommercial assistive feeding robots have already been developed to support individuals with upper limb impairments [8-11]. Examples include My Spoon (SECOM Co, Ltd) [12], SnackBot (Carnegie Mellon University Robotics Institute) [13], the Assistive Robotic Manipulator (Exact Dynamics Ltd) [14], Obi Robot (DESiN LLC) [15], iEat (Assistive Innovations) [16], and Bestic (Camanio Care AB) [17]. Table 1 outlines the key advantages, limitations, and available quantitative evidence for 7 commercial or research-based assistive feeding systems. While some platforms offer user-friendly features such as switch-based control or teach modes, most lack real-time adaptation, user-state sensing, and robust bite-delivery evaluation. The table highlights the current gaps in autonomy, sensor integration, and evidence-based validation across devices. These are predominantly passive, relying on fixed routines with limited adaptability [18]. Therefore, they struggle with delayed user responses, varied food types, and environmental challenges such as poor lighting or plate movement.

**Table 1.** Comparative analysis of existing assistive feeding robots.

Robot name and autonomy	Key advantages	Key disadvantages	Quantitative evidence
My Spoon [12]	Offers automatic, semiautomatic, and manual modes (5-DOF^a^ arm)	Requires joystick input Uses fixed mouth-position routines—no real-time adaptation	None
SnackBot [13]	Mobile platform delivers snacks in human spaces (human-robot interaction)	Not designed for bite-by-bite feeding assistance Lacks user-state feedback	The platform was never evaluated for bite delivery or self-feeding
ARM^b^ (iARM, Exact Dynamics; JACO arm, Kinova) [14]	Versatile arm mounts on wheelchairs. Performs multiple ADLs^c^ (eating, drinking, and manipulation)	No autonomous feeding intelligence Lacks specialized sensors and adaptive control	79% of 31 users completed all 16 movements 93% completed a 6-task subset Bite success not documented
Obi Robot [15]	Lightweight, compact tabletop feeder Simple switch interface, teach mode, 4-bowl food choice	Must be manually taught spoon-mouth position Cannot sense user readiness	Speech-interface pilot (n=11) showed positive usability No bite delivery data reported
Bestic [17]	4-DOF arm with rotating bowl (one-button semiautonomous control)	Uses fixed preset motions No real-time adjustment or user-state feedback	Focuses on design philosophy and user perceptions.
Meal Buddy [19]	Bowl-edge scraper removes excess food (3-DOF)	Executes preset feeding sequences only Lacks force and vision feedback	Pilot (n=3 able-bodied): mouth detection accuracy of 73%, 67%, and 52%. No bite delivery recorded
Mealtime Partner [20]	Rotating plate and mechanical spoon lift (one-button bite delivery)	Static delivery pattern No sensing of user state or environment	Historical evaluations found high abandonment Slower than human assistance

a DOF: degree of freedom.

b ARM: Assistive Robotic Manipulator.

c ADL: activities of daily living.

Various researchers have aimed to improve adaptability and intelligence through sensing and control strategies. Predictive models such as hidden Markov models have been used to estimate bite timing based on social cues and food characteristics, achieving timing errors of 1.57 seconds [7]. However, these systems often operate in open-loop and cannot adjust if the user hesitates. Similarly, support vector machines and convolutional neural networks (CNNs) [21,22] have been employed to locate bites on a plate, but they also lack real-time feedback.

More advanced systems have integrated vision and control frameworks. For instance, Mashrur et al [23] used a Personal Robot 2 (PR2; Willow Garage) equipped with faster region-based CNN and red green blue–depth (RGB-D) cameras, achieving 93% precision and an 82.8% food delivery success rate. A study by Park et al [8] deployed PR2 with a GUI interface and model predictive controller, enhancing arm safety and anomaly detection, though food handling precision remained a challenge. Hybrid controllers, such as an adaptive neuro-fuzzy inference system–proportional integral derivative controller [24] and fuzzy logic-based inverse kinematics (IKs) [25], have improved control smoothness and efficiency, but they lack real-time mouth-state feedback.

Artificial neural networks have also been applied to solve IKs [26], and low-cost platforms, such as 3D-printed arms with facial recognition [27], have broadened accessibility. However, these systems continue to rely on scripted motions. Studies conducted by Serrezuela et al [28], Mystkowski et al [29], and Gilca [30] demonstrate promise in trajectory tracking and facial landmark detection; however, sensor fusion and robustness under environmental variation remain limited. Simulation tools [31] and digital twins [32] have explored virtual training and control evaluation, but fall short of real-world responsiveness. Together, these limitations highlight the need for a fully adaptive, closed-loop feeding system that anticipates user intent, verifies safe delivery, and dynamically adjusts in real-time. To meet this need, we present a novel autonomous feeding robot that integrates time-series forecasting, machine learning, and multisensor feedback to deliver personalized, safe, and efficient feeding assistance. Our system combines autoregressive integrated moving average (ARIMA) models (to capture linear trends) with a feed-forward neural network (FFNN; to learn nonlinear behaviors), enabling real-time predictions of mouth readiness. Data from vision-based mouth detection, ultrasonic distance sensing, and strain-gauge force feedback closes the loop, allowing the robot to respond to delayed reactions, variable food textures, lighting changes, and plate movement. This study advances assistive feeding robotics through five key contributions:

1. Multimodal sensing for safe, precise delivery: integrating computer vision, ultrasonic, and force sensors ensures accurate and gentle food placement.
2. Hybrid predictive control: fusing ARIMA and neural networks enables real-time prediction of user behavior.
3. Closed-loop adaptation: a unified control loop compensates for user delays and environmental variability.
4. Demonstrated performance gains: our system improved feeding success by 15% and reduced response time by 28% over baseline models.
5. Toward truly adaptive feeding: this study lays the foundation for a learning-driven platform that supports independent eating for individuals with motor impairments.

## Methods

### Robotic Arm Hardware and Control System

**Figure 1.**
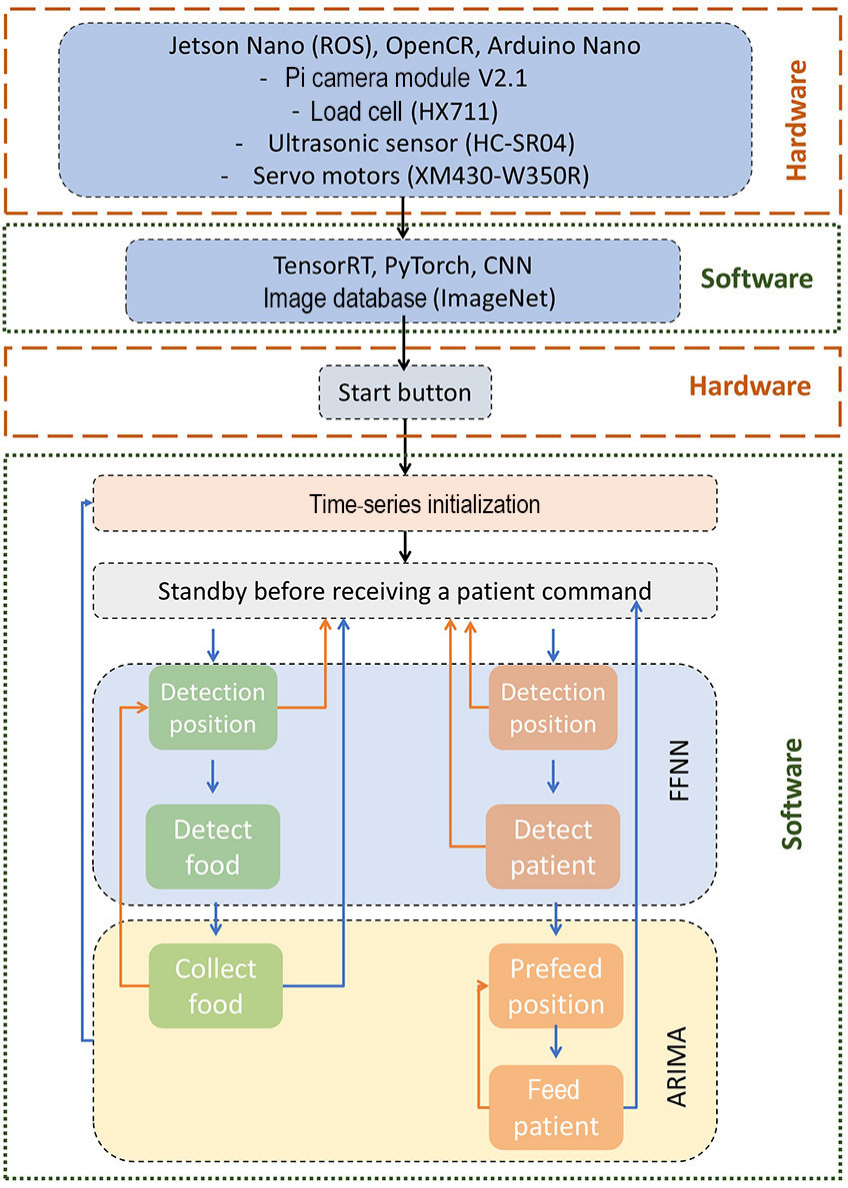
System architecture of the adaptive feeding robot. ARIMA: autoregressive integrated moving average; CNN: convolutional neural network; FFNN: feed-forward neural network; ROS: Robot Operating System.

The assistive feeding robot in this study is built around a 4-degree-of-freedom open manipulator, powered by 4 XM Series servo motors. It is equipped with a Raspberry Pi Camera Module 2 for vision, an HC-SR04 ultrasonic sensor for distance measurement, and a load cell to monitor force. The HX711 amplifier reads signals from the load cell and transmits them to an Arduino Nano, which then forwards the data to a Jetson Nano microcontroller (Figure 1).

To enable efficient communication between sensors and the control logic, a lightweight WebSocket server is hosted on the Jetson Nano, a compact artificial intelligence (AI)-enabled edge computing device with a built-in GPU. The server allows continuous, 2-way data exchange between the camera, ultrasonic sensor, and load cell, and the decision-making module that directs the robotic arm. WebSockets ensure low-latency updates, allowing the system to adjust movements dynamically in response to user behavior. For simulation and real-time testing, a digital twin of the robot was developed in Unity using its networking application programming interfaces (APIs). The Jetson Nano uses the *WebSockets* library of Python (Python Software Foundation) to run the server, while Unity serves as the client via the Native WebSocket library, maintaining synchronized sensor and actuator states between the virtual and physical environments. The Jetson Nano also runs the Robot Operating System (ROS), which manages the robot’s control architecture. NVIDIA TensorRT was used to accelerate the inference of neural networks, while the *PyTorch* library was used to train object detection and classification models.

### Feeding Simulation Scenarios in Unity

Four scenarios are simulated in Unity to test the control algorithms for the assistive feeding robot.

#### Scenario 1: Standard Operation

The robot operates under normal conditions without external disturbances or unexpected user behavior to measure the success rate of food delivery, timing, and force accuracy. The parameters are (1) feeder distance to plate and the mouth (approximately 10 cm; near), (2) distance to mouth (medium), (3) lighting (standard indoor lighting), (4) user behavior (opens mouth as the feeder gets within 15 cm), and (5) environmental stability (no movement of the table or plate).

Textbox 1. **Input and time-series decomposition.**

**Parameters**

- Degree of freedom (DOF): for the robotic arm (default=4)
- y_t−k:t_: past states of the robotic arm in time window t−k to t
- strain_t−k:t_: strain gauge data for the same time window
- sonar_t−k:t_: sonar sensor data for the same time window
- camera_t−k:t_: camera data (.eg, user mouth open or closed)
- u_t_: optional user input or manual adjustments

**Step 1: initialization**

- Set the DOFs for the robotic arm (DOF=4).
- Initialize the feed-forward neural network model with multiple layers to extract temporal features.
- Define static parameters (such as arm configuration or user preferences)

**Step 2: input**

- Collect past states of the arm: y_t−k:t_ = {y_t−k_, . . . , y_t_}
- Collect strain gauge data: strain_t−k:t_
- Collect sonar data: sonar_t−k:t_
- Collect camera data: camera_t−k:t_
- (Optional) receive user input u_t_ for manual adjustments or feedback

**Step 3: time-series decomposition**

- Decompose past arm states, strain gauge, and sonar data into trend, seasonality, and residuals: trend, seasonality, residual = decompose(y_t−k:t_,strain_t−k:t_, sonar_t−k:t_)

**Step 4: autoregressive integrated moving average (ARIMA) model**

- Fit an ARIMA model to the residuals of the decomposed data: ARIMA_model ← ARIMA(Residual)
- Use the ARIMA model to predict the next state for the residual component

#### Scenario 2: Delayed User Response

The user delays opening their mouth after the robot delivers the food, testing the system’s ability to adapt to user behavior. The parameters are (1) distance to plate (near), (2) distance to mouth (near), (3) lighting (standard indoor lighting), (4) user behavior (opens mouth, approximately 1-2 s late), and (5) environmental stability (no movement of the table or plate).

#### Scenario 3: Low-Light Conditions

The system operates in low-light conditions, testing the robustness of the object detection algorithm. Assess object detection accuracy, success rate, and timing under reduced visibility. The parameters are (1) distance to plate (medium), (2) distance to mouth (medium), (3) lighting (dim indoor lighting; approximately 50 lux), (4) user behavior (opens mouth as expected), and environmental stability (no movement of the table or plate).

#### Scenario 4: Dynamic Environment

The table or plate moves slightly during the robot’s operation, simulating a dynamic environment, and testing the system’s ability to adapt to real-time changes in plate position using the sensor’s feedback and ensure successful food delivery. The parameters are (1) distance to plate (medium), (2) distance to mouth (far), (3) lighting (standard indoor lighting), (4) user behavior (opens mouth as expected), and (5) environmental stability (plate shifts, 2-3 cm randomly)

#### Object Detection and Classification

The robot’s vision system relies on state-of-the-art object detection techniques, leveraging large datasets and advanced frameworks. ImageNet, a large-scale image database organized according to the WordNet hierarchy, was used for visual object recognition. The system is trained to detect key objects, such as tables, plates, faces, mouths, and various fruit types (including apples and strawberries). The ImageNet Large Scale Visual Recognition Challenge (ILSVRC) dataset was combined with CNNs for further training.

Since existing datasets were not sufficient for the purpose (specific tasks), transfer learning was applied. A pretrained model was fine-tuned using a custom dataset manually labeled by the research team. The custom dataset was trained on the Jetson Nano, with models preloaded with 1500 objects automatically downloaded during the build process. The system compares real-time captured images with a prebuilt database of reference images, taken under diverse lighting conditions and orientations. This ensures robust and accurate detection of objects and states, even in dynamic environments.

### Predictive Modeling Using ARIMA and FFNN

Data from sensors generates time-series interactions, serving as inputs for ARIMA (autoregressive integrated moving average) and FFNN predictions. These predictions guide the robotic arm in achieving precise movements and actions. Time-series decomposition is applied to past states and sensor data to isolate trends and residuals, with the ARIMA model predicting the residual component that captures linear relationships. The FFNN extracts features from the time-series data, learning nonlinear relationships and temporal patterns.

### Fusion of Time Series Decomposition With ARIMA and FFNN

To integrate ARIMA into the time-series decomposition framework, the residual component *R*_*t*_ was modeled with ARIMA. FFNN was used to capture nonlinear dependencies and refine predictions. The mean squared error (MSE) and *R*² metrics were calculated to evaluate the model’s accuracy, yielding a combined model MSE of 0.008 and an *R*^2^ of 94%.

We first decompose the time series into trend (*T_t_*), seasonality (*S_t_*), and residual (*R_t_*), and then apply ARIMA to model the residuals. This is enhanced by introducing the FFNN to learn deeper, nonlinear relationships from the interaction data.


(1) $$Y_{t}=T_{t}+S_{t}+R_{t}$$

where *Y_t_* is the observed interaction data at time *t* (eg, feeding times and user preferences), *T_t_* is the trend component (long-term changes in user interaction), *S_t_* is the seasonal component (cyclical patterns in user behavior), and *R_t_* is the residual component (remaining patterns or noise after removing trend and seasonality).

The residuals *R*_*t*_ are modeled using the ARIMA model:


(2) $$\begin{array}{rl} R_{t}= & \varphi_{1}R_{t-1}+\varphi_{2}R_{t-2}+\cdots +\varphi_{p}R_{t-p}\\ +\vartheta_{1}\varepsilon_{t-1}+\vartheta_{2}\varepsilon_{t-2}+\cdots +\vartheta_{q}\varepsilon_{t-q}+\varepsilon_{t} \end{array}$$

where *φ*_1_*, . . . , φ_p_* are the parameters for the autoregressive part, which models the dependency of the residuals on their past values; *θ*_1_*, . . . , θ_q_* are the parameters for the moving average part, which models the dependency of the residuals on past forecast errors (shocks or noise); *ε_t_* is the white noise (random error term at time *t*); *p* is the order of the autoregressive part; *q* is the order of the moving average part; and *d* is the degree of differencing to make the series stationary (integrated part of ARIMA).

The residuals *R_t_* can also be processed using an FFNN to further refine the predictions by capturing non-linear and complex patterns:


(3) $$R_{t}^{\text{FFNN}}=\text{FFNN}(R_{t-1},R_{t-2},\ldots ,R_{t-k})$$

where FFNN(*R_t−_*_1_*, . . . , R_t−k_*) represents the application of the FFNN model on the sequence of past residuals, *R_t−k_* to *R_t−_*_1_ for feature extraction and nonlinear modeling, *R*^FFNN^ is the refined residual prediction after applying the FFNN model, and *k* is the size of the window of past residuals used as input to the FFNN.

The final combined model is as follows:


(4) $$\begin{array}{rl} Y_{t}= & T_{t}+S_{t}+\left(\varphi_{1}R_{t-1}+\cdots +\varphi_{p}R_{t-p}\right)\\ +\left(\vartheta_{1}\varepsilon_{t-1}+\cdots +\vartheta_{q}\varepsilon_{t-q}+\varepsilon_{t}\right)+R_{t}^{\text{FFNN}} \end{array}$$

In this framework, the ARIMA model captures the remaining structure in the residuals *R_t_* after removing trend and seasonality, and the FFNN further refines this by learning nonlinear dependencies.

### Adaptive Learning and Performance Optimization

#### Simulation Setup and Evaluation Procedure

Unity simulations were executed over 150 iterations to assess the system’s learning curve. At each iteration, the robot’s internal model is updated based on the newly generated data. Approximately 1000 feeding simulations were conducted in Unity’s game engine to fine-tune these movements. The Unity environment allowed precise control over variables, enabling reproducible testing of different scenarios. MSE and *R*^2^ statistical metrics were computed to evaluate model performance. ANOVA was used to compare success rates and timing accuracy across scenarios.

#### Fuzzy Logic System

A fuzzy logic system was integrated to control the feeder fork’s actions based on the distance (ultrasonic data), fruit type (camera), force (load cell), and angle (stepper motor). The following rules are: (1) rule 1: if the distance to the plate is near and the distance to the mouth is medium or far, then apply normal stabbing force based on the fruit type; (2) rule 2: if the distance to the plate is medium and the distance to the mouth is near, then reduce the force to avoid overshooting; and (3) rule 3: if the distance to the plate is far, then do not proceed with the stabbing action (safety rule). If the fruit type is apple and the distance to the plate is near, then high force (20‐30*N*) is applied with a steep angle (approximately 60*^◦^*) for better penetration. If the fruit type is strawberry and the distance to the plate is near, then a low force (5‐10*N*) with a shallow angle (approximately 45*^◦^*) is needed to prevent damage.

A fuzzy logic system regulated the force and angle adjustments based on food type and distance. First, if the distance to the plate is near, the distance to the mouth is medium or far, and the fruit type is apple, then high force (20‐30*N*) is applied with a steep angle (60*^◦^*). Second, if the distance to the plate is near, the distance to the mouth is near, and the fruit type is strawberry, then a low force (5‐10*N*) is applied with a shallow angle (45*^◦^*).

The operational block diagram of the feeding robot is shown in Figure 1. It illustrates the integration between the hardware components (represented by orange dashed boxes) and the software modules (represented by green dashed boxes). The top hardware block includes the Jetson Nano, OpenCR, and Arduino Nano, interfaced with the Pi Camera Module V2.1, HX711 load cell, HC-SR04 ultrasonic sensor, and XM430-W350R servo motors. These components collect real-time sensory data to inform feeding decisions. The software stack comprises TensorRT, *PyTorch*, and a CNN trained on ImageNet, which supports object detection and feature extraction. The control loop is triggered via a hardware start button, followed by time-series initialization and standby mode. Two predictive models, FFNN and ARIMA, work in tandem to drive the feeding logic. The FFNN handles real-time object and face detection tasks (left: food pipeline; right: user monitoring), while ARIMA governs motion planning for food collection and patient feeding. This closed-loop architecture allows the robot to adapt to user behavior dynamically, based on synchronized input from all sensory sources.

**Figure 2.**
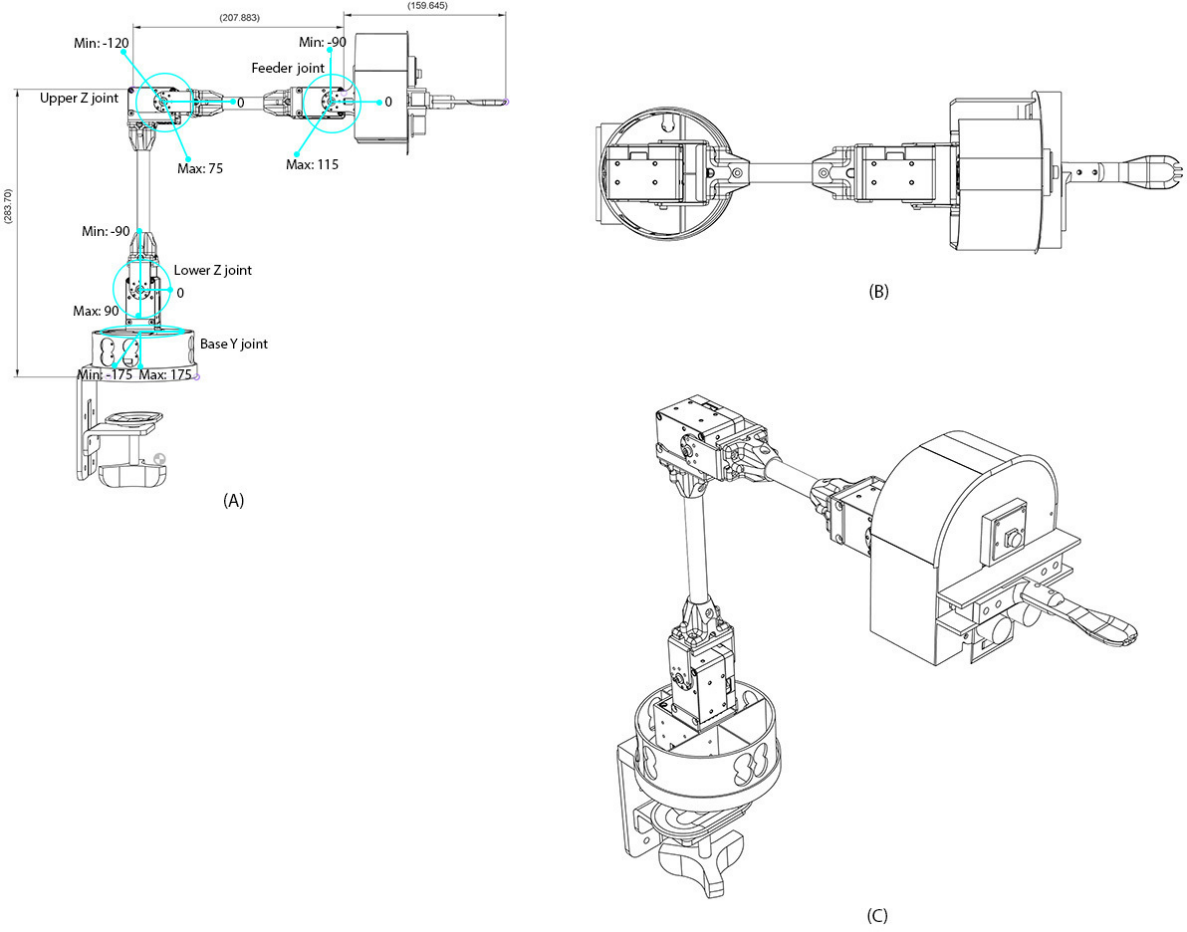
Design and configuration of the assistive robotic feeding arm. (A) 2D schematic of the robot arm showing joint labels, rotational limits, and key dimensions, including the base Y joint, lower and upper Z joints, and the feeder joint. (B) Top-down orthographic view of the robotic arm layout for spatial footprint analysis. (C) 3D perspective rendering of the full robotic assembly illustrating joint orientation and end-effector positioning for feeding tasks.

The robot operates in 2 primary sequences: scooping and forking, represented by the green blocks, and feeding, represented by the orange-colored blocks. Sequence planning is illustrated using blue arrows for “valid detection” and orange-colored arrows for “error detection” at each step in the process. The block diagram also highlights the implementation of the FFNN in the detection steps and the ARIMA model in the positioning steps. The robotic arm is equipped with joint encoders and a force sensor to generate torque commands that achieve the desired end-effector positions and orientations. Figure 2 illustrates the hardware setup and schematic architecture of the feeding robot, along with its dimensions. Figure 2A is the top view of the robotics arm, Figure 2B is the top view and Figure 2C is the 3D image with a corner view. A control system based on IK equations to execute movements was required for forking food. It includes the detailed mechanical drawings of the robot arm’s structure and joint limits. The labeled schematic highlights each degree of freedom of the arm’s mechanical range and physical configuration.

The controller system was implemented on the physical robot using an open-source ROS. The system’s physical components were fabricated using 3D printing and computer numerical control machining. The camera provided a direct line of sight for monitoring the food on the plate and the user’s mouth.

### Ethical Considerations

This study was conducted in accordance with ethical guidelines and received approval from the Anglia Ruskin University Research Ethics Committee (approval: ETH2425-0342). Participant information and consent forms were obtained before their involvement in the study. This study exclusively used simulated environments and datasets. The decision to avoid live participants was made to prioritize safety and ensure reproducibility during the robot’s development phase. Future work may incorporate user trials following ethical approval from the National Institute for Health and Care Research.

## Results

**Figure 3.**
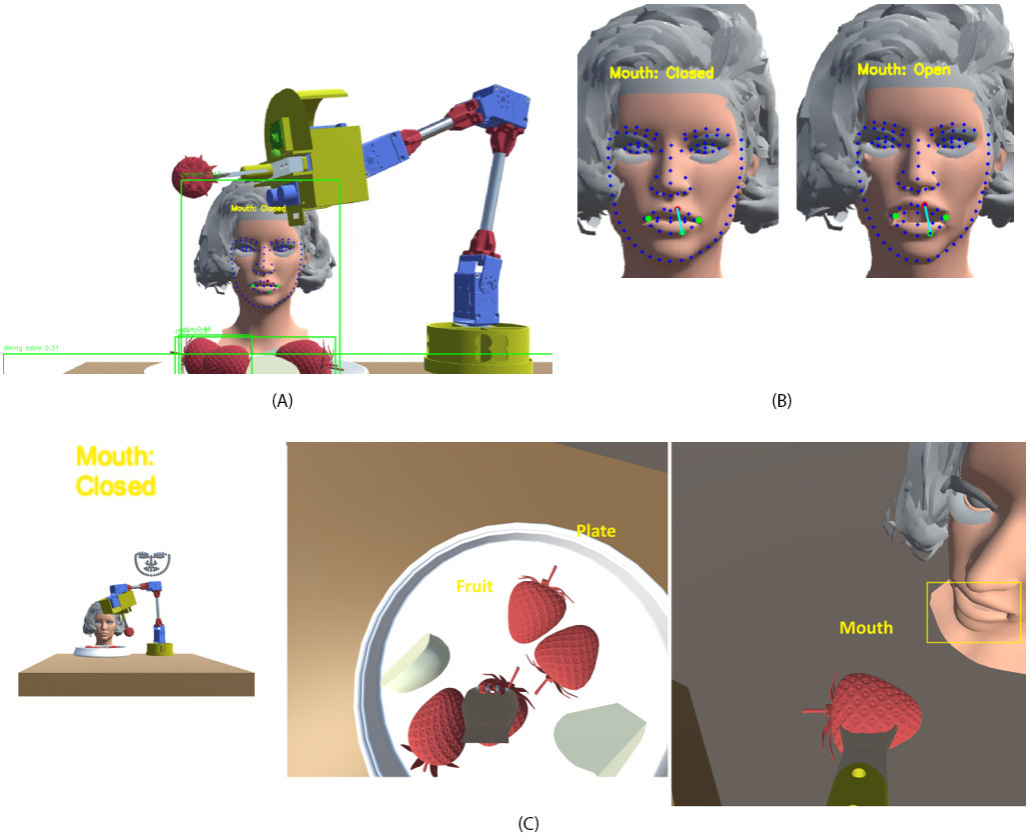
Simulated robotic feeding scenario in Unity. (A) The assistive robot detects the user’s head, mouth, and food position in 3D space using bounding boxes and landmark features. (B) Real-time mouth state detection, achieved through facial landmarks, distinguishes between open and closed mouth states to determine feeding readiness. (C) Task environment visualization: left, a full scene of robot-user interaction showing “Mouth: Closed” status; center, top-down view of the plate with multiple food items; right, bite delivery scene with mouth proximity detection using bounding box alignment.

After powering on the robot, an initialization subroutine begins, using stored positioning memory, ARIMA optimizations, and FFNN refinements. At each step during the robot’s operation, sensory data were used as training data for the FFNN and ARIMA models to enhance the patient experience further. The trained neural network was then used to initialize subsequent robot startup sequences. Figure 3A shows the front view of the primary camera in Unity, and Figure 3B highlights the facial landmarks and distances between points on the face to identify whether the mouth is open or closed. Figure 3C presents 2 perspectives: one from the primary camera and the other from the feeder camera, illustrating the integration of the object detection algorithm.

The combined ARIMA and FFNN models predict the next system state to optimize portion size and timing while also handling sensor-based adjustments. The strain gauge adjusts pressure for stabbing or scooping food, the sonar sensor adjusts position and timing based on user proximity, and the camera ensures the robotic arm only operates when the user’s mouth is open. This real-time camera feedback enhances safety and efficiency by adjusting feeding dynamics based on sensor inputs, optimizing the overall feeding process. Recurring use of the robot by the same patient can improve response time, reduce delays in sequence execution, and smooth the robot’s kinematics.

The performance of the feeding robot was evaluated across 4 simulated scenarios, with a focus on success rates, timing accuracy, and force application. The combined ARIMA and FFNN models demonstrated significant improvements in feeding accuracy and personalization. Object detection, adaptive learning, and statistical validations were conducted to illustrate system robustness.

Table 2 summarizes the robot’s performance metrics. Scenario 1 achieved the highest success rate (95%) and the lowest response time (1.5 s), while scenario 4 demonstrated adaptability to dynamic environments with a success rate of 80%. ANOVA revealed significant differences in success rates across scenarios (*F*_3,146_=12.34; *P*=.002), with scenario 1 (mean success rate of 95%, SD approximately 3.2%) outperforming scenario 3 (mean success rate of 85%, SD approximately 4.1%; *P*=.030) and scenario 4 (mean success rate of 80%, SD approximately 5.0%; *P*=.010).

**Table 2.** Average performance metrics across scenarios

Metric	Scenario 1, mean (SD)	Scenario 2, mean (SD)	Scenario 3, mean (SD)	Scenario 4, mean (SD)
Success rate (%)	95 (approximately 3.2)	90 (approximately 3.8)	85 (approximately 4.1)	80 (approximately 5.0)
Timing accuracy (s)	1.5 (approximately 0.21)	2.0 (approximately 0.32)	2.5 (approximately 0.41)	2.8 (approximately 0.48)
Force application (N)	25 (2)	20 (4)	18 (4)	22 (5)

The four scenarios simulated in Unity to test the control algorithms for the assistive feeding robot are as follows:

1. Standard operation (scenario 1): the feeding robot demonstrated optimal performance, achieving the highest success rate of 95%. The average response time was 1.5 (SD 0.21) seconds, and the force applied during the feeding process was exact, remaining within 2 N of the desired value. This scenario establishes the robot’s baseline capabilities under normal conditions.
2. Delayed user response (scenario 2): the robot showcased adaptability by adjusting its actions when the user delayed opening their mouth by 1 to 2 seconds. While the success rate decreased to 90% due to delayed feedback, the response time increased to 2 seconds compared to the standard operation. These results highlight the robot’s ability to manage user-specific behavior variations effectively.
3. Low lighting conditions (scenario 3): the object detection system experienced a slight reduction in precision and recall under dim lighting conditions (approximately 50 lux). Consequently, the success rate dropped to 85%, and the response time increased to 2.5 seconds, reflecting the challenges posed by reduced visibility. Despite these limitations, the system maintained a reasonable level of performance, demonstrating robustness.
4. Dynamic environment (scenario 4): the robot successfully adapted to plate movements of 2 to 3 cm, showcasing its real-time recalibration capabilities. However, this scenario had the lowest success rate at 80% and the highest response time of 2.8 seconds due to increased task complexity. These results emphasize the robot’s ability to handle dynamic and unpredictable conditions.

Table 3 presents the object detection metrics of the system, including precision, recall, and confidence, across key tasks. For example, the plate detection precision was 95%, while the face detection precision reached 97%. These metrics ensure accurate feeding operations, enhancing user safety and satisfaction.

**Table 3.** Object detection metrics

Object	Precision (%)	Recall (%)	Confidence (%)
Plate	95	93	90
Face	97	96	92
Open mouth	92	91	89

The combined ARIMA and FFNN model significantly outperformed standalone ARIMA and FFNN models. It achieved the lowest MSE (0.008) compared with 0.015 and 0.012, respectively, and the highest *R*^2^ (94%), relative to 85% and 88%, respectively. These results indicate the superior capability of the hybrid model to capture user interaction dynamics.

A fuzzy logic system was used to regulate the force and angle for food forking. Inputs included plate distance (ultrasonic), fruit type (camera classification), and resistance (load cell). Rules were defined for different fruit types:

- Apple: high force (20-30 N), steep angle (approximately 60°). During operation, the system measured an average applied force of 24 N (SD 1 N) and an angle of 59° (SD 1°), aligning closely with expectations.
- Strawberry: low force (5–10 N), shallow angle (approximately 45°). The measured force was 7 N (SD 0.5 N), with an angle of 44° (SD 1°), ensuring minimal fruit damage and controlled delivery.

Force and angle adjustments were verified by comparing expected versus measured values recorded by sensors during the operation.

**Figure 4.**
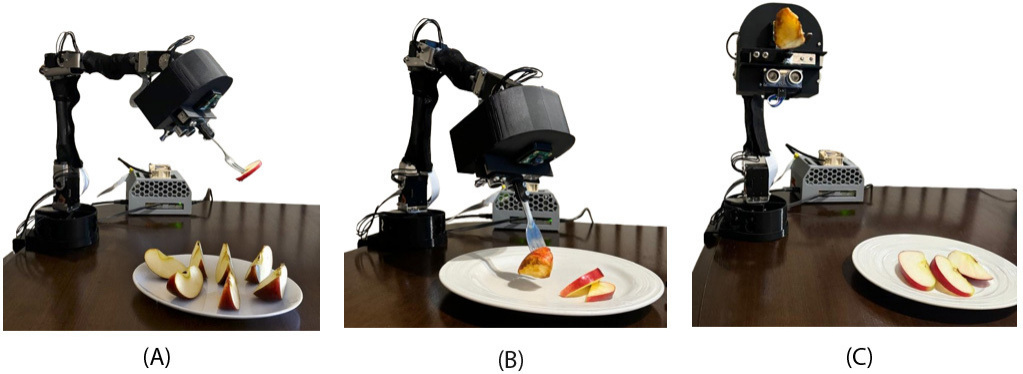
Operational sequence of the assistive feeding robot during a food delivery task. (A) The robotic arm localizes and targets a food item (apple slice) for acquisition. (B) The robot successfully pierces and lifts the apple slice using a fork-like end-effector. (C) The arm moves the food toward the user’s mouth position, demonstrating autonomous bite delivery.

Figure 4 illustrates the robotic arm in a real-world setting while performing food targeting, picking, and delivery tasks. In Figure 4A-B, the apple is successfully forked and picked up, showcasing the effectiveness of the stabbing mechanism. Finally, Figure 4C shows the robotic arm delivering the food to a designated mouth position. This demonstrates the robot’s operational workflow, covering critical steps of targeting, stabbing, and delivering food to a target. The experiment under scenario one yielded an average accuracy rate of 87% over 50 iterations, emphasizing the system’s reliability and consistency.

**Figure 5.**
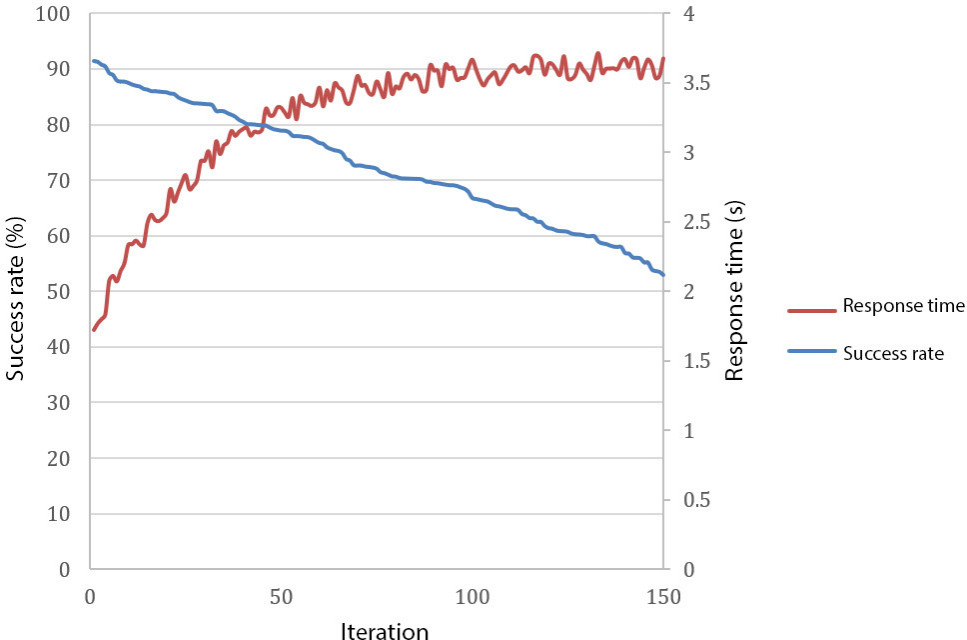
Performance improvements across iterations. The red line represents the success rate over 150 iterations. The blue line represents the reduction in response time over iterations.

Figure 5 illustrates the iterative improvements in success rate and response time over 150 iterations in the fourth scenario of the simulated environment. Key parameters analyzed included stabbing angles, applied forces, and success rates for apples, demonstrating the robot’s adaptability to varying conditions. It showed a steady improvement in success rate, starting at approximately 40% in initial iterations and stabilizing at 90% after 90 iterations. This progression underscores the effectiveness of the adaptive learning approach in optimizing performance. Figure 5 also illustrates the corresponding reduction in response time, which starts at 3.6 seconds and improves to 2.2 seconds (*P*<.001), highlighting enhanced efficiency.

**Figure 6.**
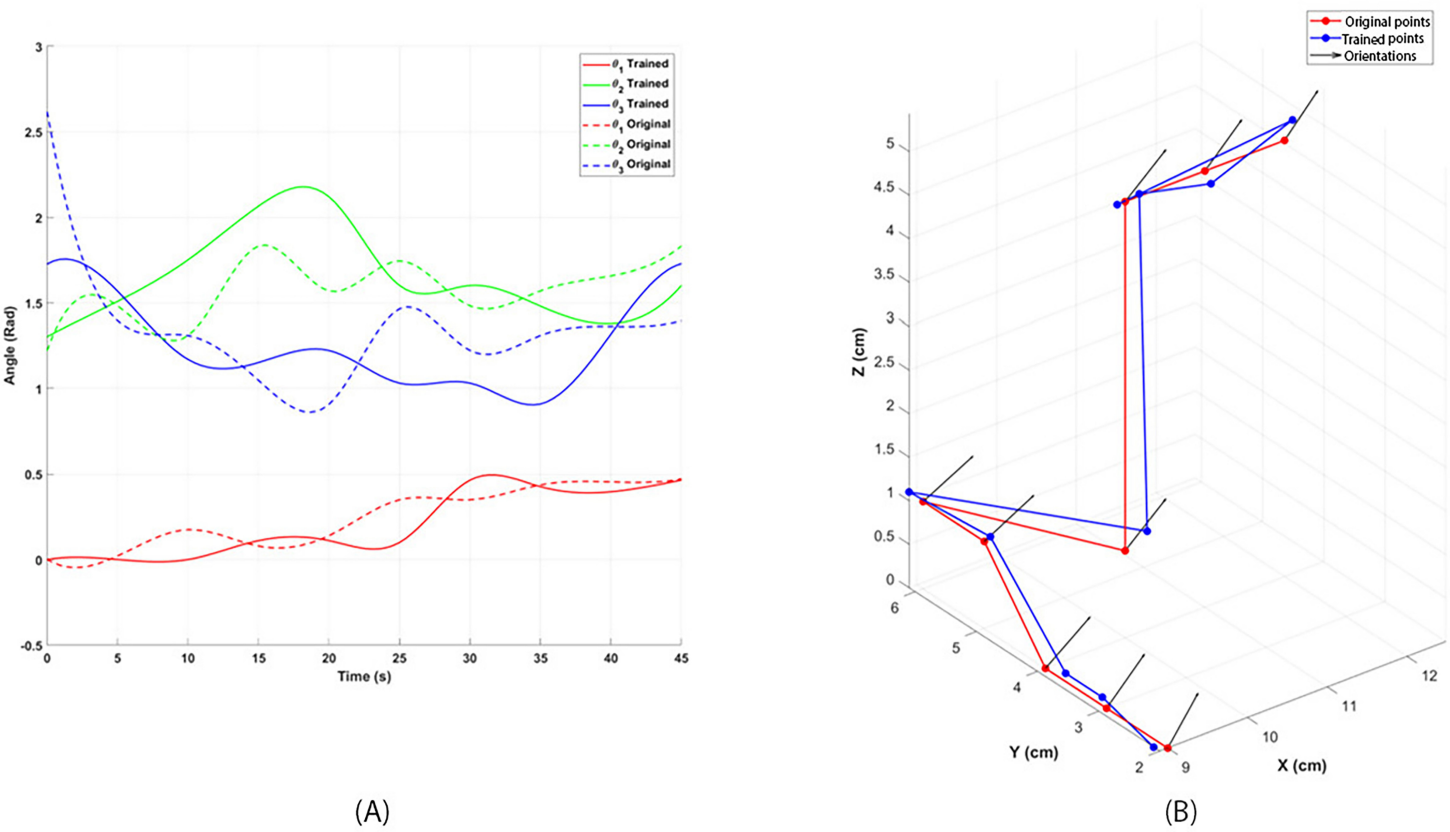
(A) Joint angle trajectories during movement execution show reduced overshooting and undershooting compared to the untrained control system. (B) The actuator position is in 3D space during the same movement, with an optimized path and control system output. The red line represents the success rate over 150 iterations, while the blue line represents the reduction in response time over iterations.

The kinematic response and stability of the robot have also been enhanced, as demonstrated in Figure 6. Figure 6A illustrates the angular movement of each joint during a specific motion sequence. In contrast, Figure 6 depicts the 3D spatial positioning of the actuator during the execution of the same movement set. Both graphs compare the optimized system’s performance to the original untrained control system output. Notable improvements in the robot’s pathfinding and control system output are evident in both graphs. In Figure 6A, there is an apparent reduction in the overshooting and undershooting of servo adjustments needed to achieve the desired joint angles, minimizing errors and enhancing precision. These refinements have significantly reduced the jerkiness in the robot’s arm movements and diminished vibrations in the final actuator. Consequently, food delivery to the user is smoother and more controlled, which underscores the improved kinematic performance and stability of the robot.

**Figure 7.**
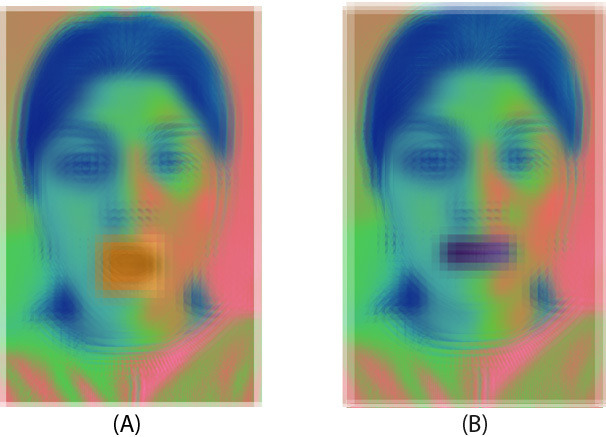
Mouth state detection using neural network image segmentation. (A) The system detects an open mouth using a region-of-interest (ROI) around the lips, highlighted in orange. (B) The system detects a closed mouth with a reduced feature response in the ROI, shown with a darker overlay. This classification supports the robot’s decision-making process for safe and timely food delivery.

Figure 7 illustrates the facial recognition and mouth detection neural networks integrated into the robot. Using advanced image recognition techniques. Using a VGG 16 convolutional network (proposed by the Visual Geometry Group at the University of Oxford [33]), the robot detects and highlights the users’ facial features. It determines if they have an open mouth or not before approaching the user. The neural network initially separates the user’s face from the background and then identifies the control volume box where the user’s mouth is located. Depending on the facial characteristics of a user, the neural network constantly updates the status of the user between mouth open and closed. This is a key feature of the robot that enables it to perform the feeding task reliably, providing a layer of system control within the feeding sequence. Whenever the user has a closed mouth, the feeding sequence is halted, waiting for either an override command or the mouth open status to continue the feeding sequence.

## Discussion

### Principal Findings

In this study, we demonstrated that a hybrid control architecture, combining time-series forecasting (ARIMA), nonlinear modeling (FFNN), and multisensor feedback, can significantly enhance the performance of an assistive feeding robot in both simulation and physical hardware. Our approach yielded a combined MSE of 0.008 and an *R*^2^ of 94%, which improved feeding success rates from 75% to 90% and reduced response times by 28% (from 3.6 s to 2.2 s), outperforming both ARIMA-only and FFNN-only controllers. These results underscore the value of fusing statistical and machine-learning techniques. ARIMA captures linear trends in a user’s eating rhythm, while the FFNN models nonlinear variations, together enabling proactive adjustment of timing and portion size. The closed-loop integration of vision-based mouth readiness detection, ultrasonic ranging, and strain-gauge force sensing further enables the system to adapt to delayed user responses, diverse food textures, low-lighting conditions, and plate movements, which are conditions under which prior passive or semiautonomous feeders struggle. Our fuzzy logic–driven force and angle controller also proved effective at tailoring grip strength to different foods (eg, apples vs strawberries), minimizing spills and enhancing safety. Compared to legacy systems like My Spoon and Obi Robot, which rely on fixed trajectories or manual “teach” modes, our robot autonomously adjusts its behavior in real-time, reducing caregiver intervention and improving user autonomy. Despite the promising results, several limitations remain and will guide future research. While the Unity simulation enabled rapid iteration and control benchmarking, it cannot fully replicate the unpredictability of real-world settings, including table height variability, user posture changes, and lighting variation.

This study focused on only 2 food types, which limits generalizability to more complex diets involving varied textures, consistencies, and utensil requirements (eg, soups or mixed meals). Although the system is designed to operate autonomously, setup and supervision may still be required. User-friendly interfaces, minimal daily calibration, and rapid onboarding for caregivers will be key to adoption. We are exploring guided setup workflows and voice-driven overrides to reduce learning curves. Furthermore, no real-user trials have been conducted, meaning aspects such as user comfort, adaptability, and long-term acceptance remain unexplored. To address these limitations, we are transitioning our control system to a physical robotic arm integrated with the proposed sensor suite. Initial bench-top experiments will assess trajectory accuracy, timing responsiveness, and force safety across a broader range of feeding conditions. Following this, a pilot study involving 3 to 5 individuals with upper-limb impairments is planned to evaluate comfort, performance, and real-world usability. By addressing deployment challenges, including safety, maintenance, training, and personalization, we aim to ensure that future iterations are not only technically robust but also clinically viable and user-friendly. These evaluations will inform iterative improvements and alignment with clinical standards. Ultimately, our goal is to deliver a safe, autonomous feeding solution that promotes independence, comfort, and dignity for individuals with motor impairments across diverse care environments.

### Limitations and Conclusions

In this work, we used simulated environments and datasets to validate an adaptive feeding robot that significantly advances the state of assistive dining technology. By integrating time-series forecasting (ARIMA), nonlinear modeling (FFNN), and multisensor feedback (vision, ultrasonic ranging, and strain-gauge force sensing), our system anticipates each user’s unique feeding pace. It dynamically adjusts both timing and force for safe, precise spoon-to-mouth delivery. In over 1000 Unity simulations and on physical hardware, the hybrid controller achieved an MSE of 0.008 (*R*^2^=94%), increased feeding success from 75% to 90%, and reduced response times by 28% (3.6 s-2.2 s*; P<.*001), outperforming ARIMA-only and FFNN-only baselines. Despite these promising results, our study has several limitations. First, performance was primarily evaluated in simulation; real-world variability, such as unstructured environments, diverse plate geometries, and spontaneous user movements, remains to be tested in live trials. Second, we focused on 2 food types (apple and strawberry); future work should encompass a broader range of textures, shapes, and portion sizes. Finally, user acceptance, comfort, and long-term usability were not assessed; ethical considerations surrounding autonomy and trust in human-robot interaction warrant further investigation. Looking ahead, we plan to conduct clinical pilot studies to evaluate real-world efficacy and user satisfaction, extend our sensor suite to include depth cameras and tactile arrays, and explore advanced forecasting methods (eg, recurrent neural networks and transformers) for even finer-grained personalization. By addressing these challenges, we aim to transition from a laboratory prototype to a robust, user-centered assistive solution, empowering individuals with motor impairments to dine independently and with dignity.

## Abbreviations

ARIMA: auto regressive integrated moving average
CNN: convolutional neural network
FFNN: feed-forward neural network
IK: inverse kinematic
ILSVRC: ImageNet Large Scale Visual Recognition Challenge
MSE: mean squared error
PR2: Personal Robot 2
RGB-D: red green blue–depth
